# 3D printed kombucha biomaterial as a tissue scaffold and L929 cell cytotoxicity assay

**DOI:** 10.1111/jcmm.18316

**Published:** 2024-05-09

**Authors:** İlkyaz Bağlan, Edagül Yanbakan, Tuğba Tuncel, Ayşe Koçak Sezgin, Emirhan Bozoğlan, Derya Berikten, Fatih Kar

**Affiliations:** ^1^ Koc University İstanbul Türkiye; ^2^ Training and Research Center Kütahya Health Sciences University Kutahya Türkiye; ^3^ Department of Pharmacognosy, Faculty of Pharmacy Anadolu University Eskişehir Türkiye; ^4^ Department of Medical Biochemistry, Faculty of Medicine Kutahya Health Sciences University Kütahya Türkiye; ^5^ Department of Medical Services and Techniques, Tavşanlı Vocational School of Health Services Kutahya Health Sciences University Kütahya Türkiye; ^6^ Department of Molecular Biology and Genetic, Faculty of Engineering and Natural Sciences Kutahya Health Sciences University Kütahya Türkiye

**Keywords:** biomaterial, fibroblast, kombucha, SCOBY, tissue scaffold, toxicity

## Abstract

Tissue engineering includes the construction of tissue‐organ scaffold. The advantage of three‐dimensional scaffolds over two‐dimensional scaffolds is that they provide homeostasis for a longer time. The microbial community in Symbiotic culture of bacteria and yeast (SCOBY) can be a source for kombucha (kombu tea) production. In this study, it was aimed to investigate the usage of SCOBY, which produces bacterial cellulose, as a biomaterial and 3D scaffold material. 3D printable biomaterial was obtained by partial hydrolysis of oolong tea and black tea kombucha biofilms. In order to investigate the usage of 3D kombucha biomaterial as a tissue scaffold, “L929 cell line 3D cell culture” was created and cell viability was tested in the biomaterial. At the end of the 21st day, black tea showed 51% and oolong tea 73% viability. The cytotoxicity of the materials prepared by lyophilizing oolong and black tea kombucha beverages in fibroblast cell culture was determined. Black tea IC_50_ value: 7.53 mg, oolong tea IC_50_ value is found as 6.05 mg. Fibroblast viability in 3D biomaterial + lyophilized oolong and black tea kombucha beverages, which were created using the amounts determined to these values, were investigated by cell culture Fibroblasts in lyophilized and 3D biomaterial showed viability of 58% in black tea and 78% in oolong tea at the end of the 7th day. In SEM analysis, it was concluded that fibroblast cells created adhesion to the biomaterial. 3D biomaterial from kombucha mushroom culture can be used as tissue scaffold and biomaterial.

## 1 INTRODUCTION

Biopolymers are natural molecules produced by living organisms. There is also a usage of biopolymers, biomaterials to fulfil the functions of living tissues in the human body. Since biomaterials are in constant contact with the human body, they must have some physical and chemical properties. Some of these properties are non‐toxic, non‐carcinogenic, non‐allergenic, long‐lasting, sterilizable and biocompatible. Biomaterials are essential for the preparation of appropriate scaffolds during tissue regeneration. However, developing a suitable scaffold with desired physicochemical and biomechanical properties is a very challenging process. One of the basic substances that should be found in biomaterials is cellulose. The main reason for using bacterial cellulose instead of plant cellulose is that it can be purified more easily. At the same time, bacterial cellulose provides better mechanical strength and crystallinity.^1^, ^2^ Structurally, bacterial cellulose is thinner and more unbranched compared to plant cellulose; this property has contributed to various properties such as better mechanical durability. Other properties such as high crystallinity, biocompatibility, non‐toxicity and high porosity make it a suitable biomaterial for tissue engineering in technological implementation in bioplastics, bioenergy, food fortification and packaging.^3^, ^4^

Tissue engineering is a field that offers solutions to problems arising from limiting factors such as donor scarcity and immune response formation, especially in organ transplantation. Although this area includes the construction of tissue or organ scaffolding, it requires tests to preserve cell viability. Today, this implementation is especially focused on three‐dimensional (3D) scaffolds. The advantage of 3D scaffolds over two‐dimensional scaffolds is that they provide homeostasis for a longer period of time, allow better imitation of real tissue, at the same time do not interfere with the functioning of other functions in the body, protect the organism from the environment by interacting with the environment, reduce the usage of animal models by being used instead of animal models, have a more realistic way to grow and treat tumour cells. Investigation of SCOBY‐derived kombucha as a scaffold material that can meet these properties was aimed in this study. Symbiotic culture of bacteria and yeast (SCOBY) is a combination of bacteria and yeasts and is a source of cellulose for biomaterials. As a result of microbial activity, SCOBY is formed at the liquid–air interface.^5^ The microbial community attached to SCOBY can be a source for subsequent kombucha production.^6^ Kombucha culture consists of yeasts such as *Saccharomyces cerevisiae*, *Saccharomyces inconspicus*, *Saccharomyces ludwigii*, *Schizosaccharomyces pombe*, *Candida tropicalis*, *Candida krusei*, *Debaryomyces hansenii*, *Brettanomyces* spp., *Kloeckera* spp., *Zygosaccharomyces bailii* ve *Zygosaccharomyces kombuchaensis*, *Torulospora* spp., *Pichia* spp., *Mycotorula* spp., *Mycoderma* spp. and acetic acid bacteria, such as *Acetobacter xylinum*, *Acetobacter xylinoides*, *Bacterium gluconicum*, *Acetobacter suboxydans*, *Gluconobacter liquefaciens*, *Acetobacter aceti* ve *Acetobacter pasteurianus* and *Lactobacillus bulgaricus* belonging to the lactic acid bacteria group^7^ Kombucha is a beverage made by fermenting sweetened tea with its mushrooms. Various types of tea such as black tea, green tea, oolong tea can be used in the fermentation of tea mushrooms. Kombucha beverage contains acids, such as acetic, gluconic and glucuronic acid. Apart from these, various sugars, water‐soluble vitamins, amino acids; it also contains biogenic amines, purines, pigments, lipids, proteins, hydrolytic enzymes, ethanol, carbon dioxide, polyphenols, D‐saccharic acid 1,4‐lactone (DSL), minerals (manganese, iron, nickel, copper, zinc, lead, cobalt, chromium and cadmium), anions (fluoride, chloride, bromide, iodide, nitrate, phosphate and sulfate) and metabolic products of yeast and bacteria. It has been reported that antioxidant activity in kombucha beverages originated from polyphenols, DSL (D‐saccharic acid‐1,4‐lactone), ascorbic acid, vitamin E and carotenoids.^8^ It has been stated that the beneficial effects of kombucha beverage may be caused by the synergistic effect between bioactive components such as polyphenols and glucuronic acid in its content.^9^ In pre‐clinical studies on kombucha, effects such as antimicrobial, antioxidant, antihypertensive, antidiabetic, anticancer, anti‐inflammatory, cholesterol‐lowering, anti‐ageing, preventive of cardiovascular and neurodegenerative diseases and liver protective have been revealed.^10^, ^11^, ^12^, ^13^, ^14^, ^15^, ^16^, ^17^

We aimed to investigate the potential of kombucha to be an implant that can be used in the treatment of various diseases, based on the usage of kombucha as a source of biomaterials and its bioactivity. It is planned to carry out the fermentation process with the usage of oolong tea and black tea. At the end of this process, it is aimed to obtain kombucha biofilms and kombucha beverage. It is purposed to obtain biomaterials from kombucha biofilms. It was designed to be used as a tissue scaffold by 3D printing of the obtained printable biomaterial. It was aimed to determine the cytotoxicity of this tissue scaffold by examining its viability in “L929 cell line (mouse fibroblast) 3D cell culture.” It was designed to investigate the adhesion status of fibroblast cells to the 3D biomaterial by SEM imaging. It is planned to determine their viability in fibroblast cell culture by adding the material obtained as a result of the removal of the water content of the kombucha beverage by lyophilization to the 3D biomaterial, which was created considering that it may have cell regenerative properties. As a result, the development of a bioimplant that may have the potential to be used for therapeutic purposes, if cell viability is achieved at desired levels, is among the future aims. It is hoped that the biomaterial to be obtained in this way can be used as a bioimplant and will provide an opportunity to solve many health problems such as meniscus lesions. This study is original and different from other studies in terms of 3D cell culture experiment, examining the effects of oolong and black tea on kombucha components, fermentation conditions, in vitro cell culture experiment conditions, kombucha beverage being similar to ECM with a biomimetic design and the lyophilized material with similar content to ECM has the potential to be an bioimplant that can be used in the treatment of various diseases by adding to the tissue scaffold.

In addition, it has been seen in the literature reviews that there is not enough literature on its usage as a 3D biomaterial. As a result of this study, it is thought that the obtained results will contribute to developing tissue engineering, artificial tissue research and bioimplant implementation.

Our hypotheses, biodegradable/pressure‐stable kombucha biofilms may be a suitable biomaterial. The scaffold obtained using Kombucha biofilms can be the attachment surface of the cells, and a suitable bioimplant can be developed on this basis. Kombucha beverage may be a source for scaffolding that can be used in various treatments with components close to ECM content.

## 2 MATERIALS AND METHODS

### 2.1 Consumables

Oolong tea, Black tea, Sucrose, Yeast extract, Peptone, Distilled water, Kombucha SCOBY culture, NaOH, Sulfuric acid, DMEM, FBS, Streptomycin + Penicillin, PBS, DNase‐RNase free water, The CellTiter96® AQueous One Solution Cell Proliferation Assay, sterile flask and petri.

### 2.2 Devices

OHAUS STARTER 3100 pH meter, Thermo Ultimate 3000 HPLC (600 Bar), zorbax eclips XDB‐C18 column, CHRIST ALPHA PLUS Lyophilizer, CO2 oven, RWD cell counter, laminar flow, automatic pipette sets, microplate reader spectrophotometer, JEOL JSM‐5600LV SEM.

### 2.3 Kombucha beverage fermentation, separation of kombucha beverages and kombucha biofilms

It was added 5 g of oolong tea to one of two separate 1 l of boiling water and 5 g of black tea to the other and waited for 10 min to brew. Afterwards, 50 g of sucrose, 5 g of yeast (yeast extract) and 0.5 g of peptone were added to each of them, and the pH was adjusted to 5 after cooling to room temperature. In this study, 10% (1 L per 100 g) of live kombucha beverage and starter mushroom disc SCOBY (200 g, 90 mm diameter) were added. It was fermented for 21 days in a place out of the sun. On the day 21, the top layer was separated from the fermentation medium by filtration. The separated layer was washed with distilled water and left to dry at room temperature. Thus, the prepared biofilms were used as pure kombucha bacterial cellulose. Kombucha beverages were transferred to separate containers. The pH of Kombucha beverage samples was determined using the OHAUS STARTER 3100 pH meter.

### 2.4 Partial hydrolysis of kombucha biofilms

Thirty per cent (30%) (V/V) sulfuric acid solution was prepared, kombucha biofilms (oolong and black tea) were kept in this solution in a 70°C water bath for 4 h to perform acid‐induced partial hydrolysis. Partially hydrolyzed samples were washed with 0.2 M NaOH for 1 h. The resulting samples were dialyzed for 1 day to turn them into a printable gel suitable for extrusion.

### 2.5 Analysis of kombucha beverages by HPLC

The analyses of kombucha beverages obtained with oolong and black tea were carried out with Thermo Ultimate 3000 HPLC (600 Bar). The samples were separated with a zorbax eclips XDB‐C18 column using 80% Milli‐*Q* water and 20% acetonitrile mobile phase as the organic phase. The flow rate is 0.75 mL/min and the total run time is 10 min. The column was operated at room temperature and the injection volume of the samples was 5 μL.

### 2.6 Lyophilization of kombucha beverages

Oolong and black tea kombucha beverages obtained as a result of fermentation were filled into 50 mL falcons and kept for 6–24 h at −80°C. When the samples were completely frozen, they were placed in the chamber of the lyophilization device. The main drying and final drying processes were set at −76°C and 0.0010 mbar pressure, and the study was carried out for 48 h. Oolong tea and black tea kombucha beverages were dehydrated by freezing in a lyophilizer for 10 days and lyophilized samples were obtained.

### 2.7 3D scaffold (Tissue Scaffold) created from kombucha biofilms L929 cell line (Mouse Fibroblast) cell culture experiment

The L929 cell line was obtained from the Alum Institute. Cells were incubated in T75 cm2 flasks in DMEM containing 10% FBS, 1% Streptomycin + Penicillin, in a humid atmosphere containing 5% CO_2_ at 37°C. It was reproduced until it reached 80% fullness. Collected cells were counted on the RWD Automated cell counting device. The prepared bio ink was transferred to 5 mL printing syringes. 25G needle tip is preferred. For the printing parameters, experiments were first made between 0 and 50 psi pressure. Since we used PLA scaffold structure in the experiment, no cross‐linking agent was used. It was printed at a speed of 10 mm/s with a pressure of 35 psi into an angled porous PLA scaffold that permeates the medium but keeps the bioink stable. Printing was carried out in a sterile environment with the Axolotl C3 bioprinter system (positive internal pressure HEPA filter system and UV sterilization). It was left to the incubation process after printing. Sterilized kombucha black tea and oolong tea biomaterial (3D Scaffold (Tissue Scaffold)) and blank cells as control were seeded into sterile petri dishes at 1 × 10^7^ cells as Day 14 and Day 21 experiments. At the end of the 14th and 21st days, the viability analysis of the materials was examined with The CellTiter96® AQueous One Solution Cell Proliferation Assay and the measurements were made at 490 nm in a microplate reader spectrophotometer (ThermoScientific, USA) in triplicate. Their viability rates were analysed by comparison with control wells.

### 2.8 Determination of cytotoxicity of lyophilized kombucha beverage samples on L929 cell line (Mouse Fibroblast)

Trypsinized cells were seeded into 96 well plates containing 2.5 × 105 cells. Cells were applied at 20, 10.5 and 1 mg doses for black and oolong tea biomaterials, examined with The CellTiter 96® AQueous One Solution Cell Proliferation Assay after 48 h and measured in triplicate on a microplate reader spectrophotometer (ThermoScientific, USA) at 490 nm.

### 2.9 Lyophilized kombucha beverage samples + 3D Scaffold (Tissue Scaffold) L929 cell line (Mouse Fibroblast) cell culture experiment

Collected cells were counted on the RWD Automated cell counting device. 3D biomaterials obtained from incubated, sterilized black tea and oolong tea and blank cells as control were seeded into a sterile petri dish at 1 × 10^7^ cells. IC_50_ values were applied to the growth medium according to the results of cytotoxicity. These values are 6.051 mg for oolong tea and 7.5 mg for black tea. At the end of the 7th day, the collected cells were examined with The CellTiter 96® AQueous One Solution Cell Proliferation Assay and the measurements were made in triplicate at 490 nm in a microplate reader spectrophotometer (ThermoScientific, USA).

### 2.10 SEM imaging of lyophilized kombucha beverage samples and 3D scaffold fibroblast cell culture samples

Oolong 3D biomaterial Day 21, Oolong 3D biomaterial and oolong lyophilized beverage 3D material Day 7; Black tea 3D biomaterial Day 21, Oolong 3D biomaterial and oolong lyophilized beverage 3D material Day 7 samples were placed at 4°C as a prefixation step for 24 h in 2.5% glutaraldehyde (prepared in 0.1 M phosphate buffer, pH 7.4). Then it was rinsed twice with 0.1 M phosphate buffer (pH 7.4), fixed for 1 h at room temperature using 1% osmium tetroxide and finally rinsed with distilled water. After that, the samples were dehydrated using gradual ethyl alcohol concentrations (30%, 50%, 70%, 90% and 96%) for 15 min and then for 30 min of absolute alcohol. The sample was dried using the critical point dryer Polaron CPD 7501 Critical Point Dryer (VG. Microtech, East Sussex, UK). Carbon conductive paint is used for assembly; Gold plating with Polaron SC7620 Sputter Coater was used for the samples. Finally, each sample was examined by using a JEOL scanning electron microscope (JEOL JSM‐5600LV). Several areas of each sample were systematically scanned.

### 2.11 Statistical methods

Data are expressed as mean ± SE of at least three independent experiments. GraphPad Prism was used for one‐way ANOVA, *t*‐test and *p* < 0.05 was considered statistically significant.

## 3 RESULTS

### 3.1 Kombucha beverage fermentation results

The kombucha disc obtained during fermentation with oolong tea weighed 83.2 g Oolong tea kombucha beverage was measured 800 mL. The kombucha disc obtained by fermentation with black tea weighed 82.3 g black tea kombucha beverage was measured 700 mL. At the end of the 21st day, the pH of oolong tea kombucha beverage was 2.88, and the pH of black tea kombucha beverage was 2.94. 3D material suitable for extrusion was obtained by partial hydrolysis of kombucha biofilms.

### 3.2 Analysis of kombucha beverages by HPLC

**FIGURE 1:**
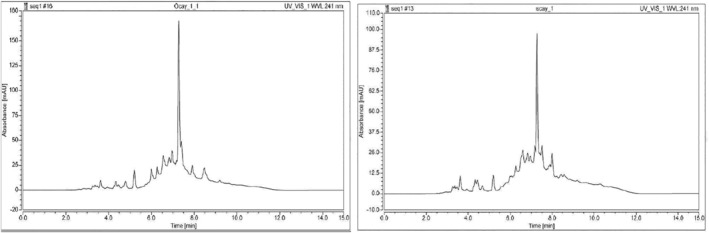
Oolong tea and black tea kombucha beverage HPLC analysis chromatogram.

The chromatogram of oolong tea kombucha beverage is shown in Figure 1A, and the chromatogram of black tea kombucha beverage is shown in Figure 1B. Since the standard substance supply conditions could not be provided, it was commented by comparing the analysis results and the literature. As a result of lyophilization of oolong tea kombucha beverage was obtained, 22.2 g lyophilized biomaterial. As a result of lyophilization of oolong tea kombucha beverage was obtained, 22.11 g lyophilized biomaterial.

### 3.3 Fibroblast cell culture with 3D Scaffold (Tissue Scaffold) created from kombucha biofilms

**FIGURE 2:**
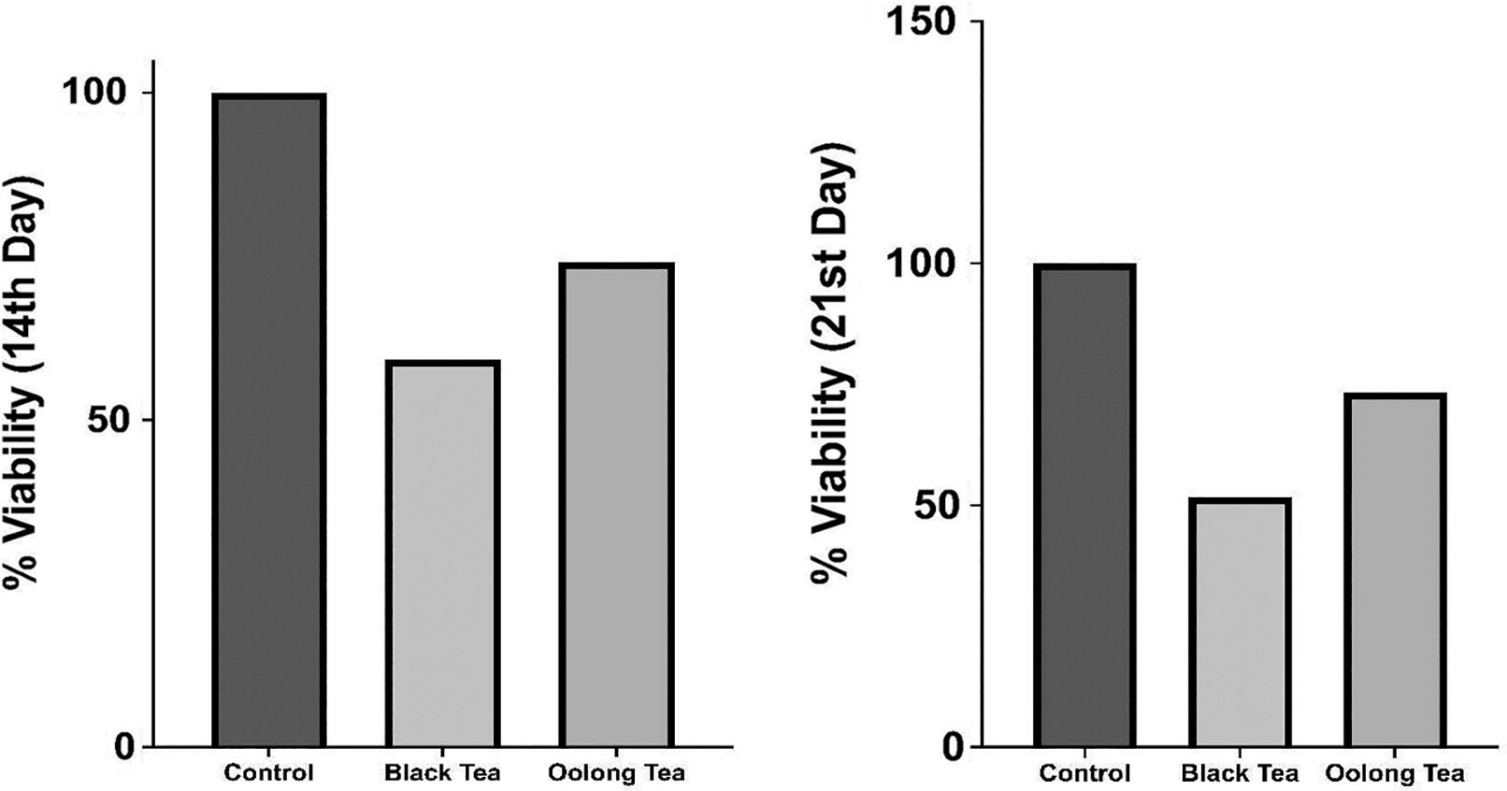
3D biomaterial day 14 and 21 results.

**FIGURE 3:**
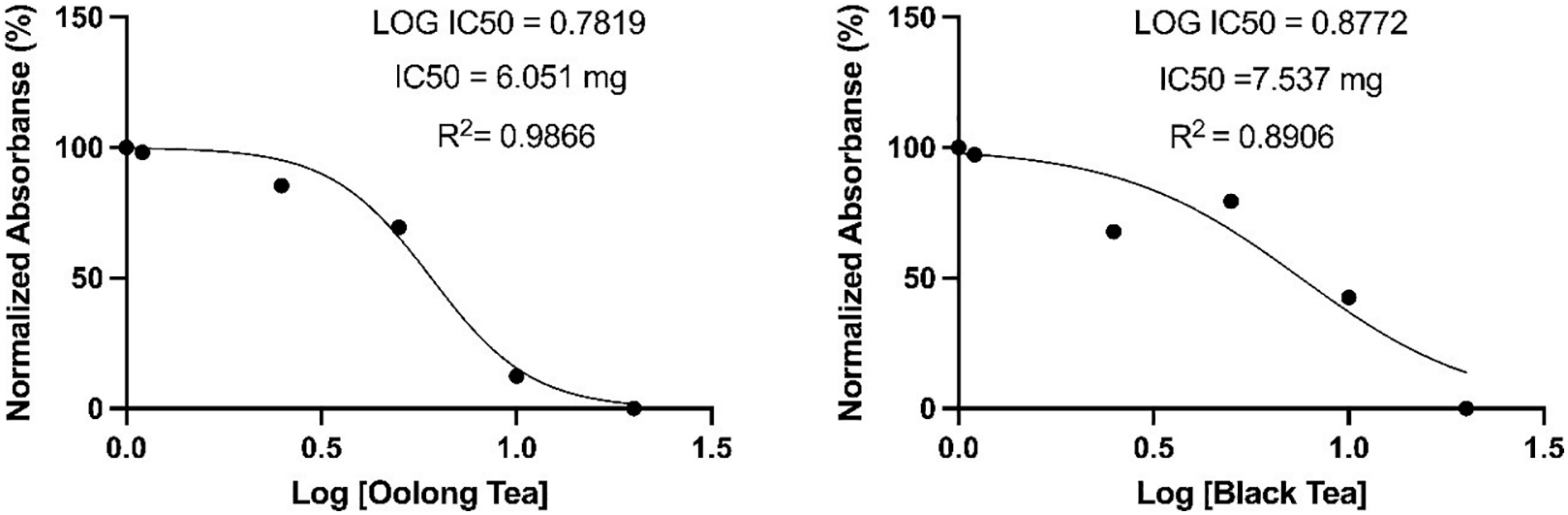
Cytotoxicity values in L929 cells of lyophilized kombucha beverage.

At the end of the fourteenth day, black tea kombucha 3D Scaffold showed 59% viability, whereas oolong tea kombucha 3D Scaffold showed 74% viability (Figure 2). At the end of the 21st day according to the analysis black tea kombucha 3D Scaffold showed viability at the rate of 51%, while oolong tea kombucha 3D Scaffold showed viability at the rate of 73%. According to the test results, the IC_50_ value for black tea was 6.05 mg and *R* ^2^ = 0.98. For oolong tea, the IC_50_ value was 7.53 mg and *R* ^2^ = 0.89 (Figure 3).

**FIGURE 4:**
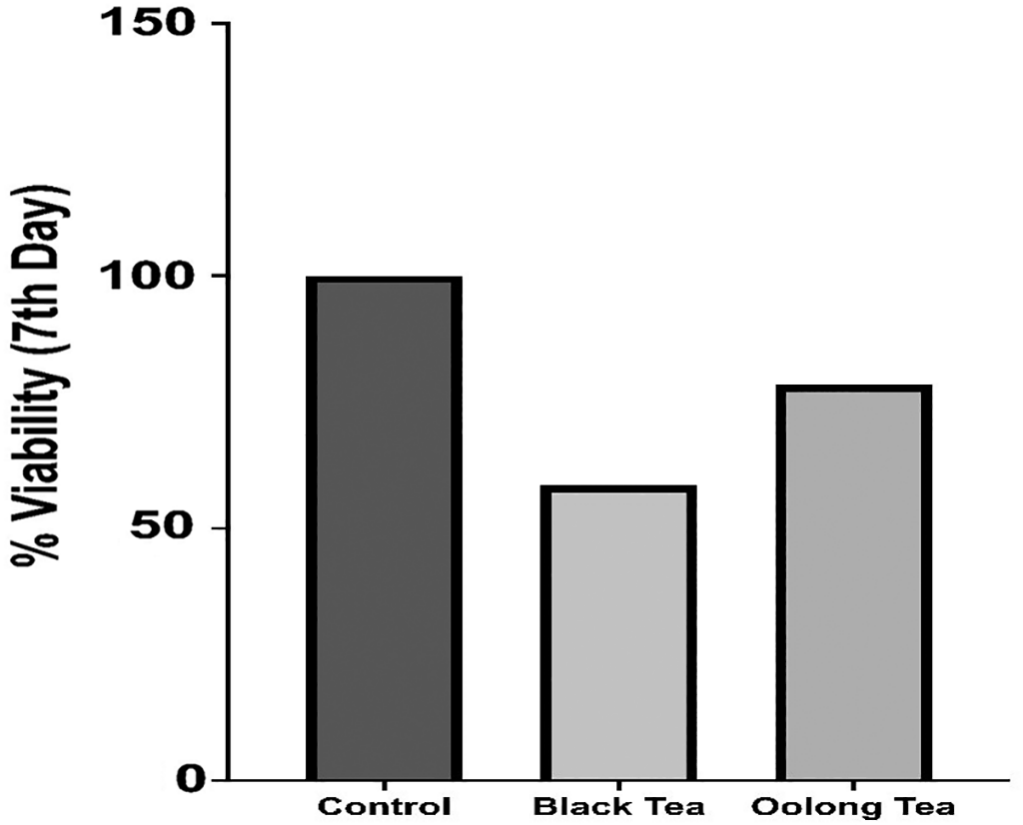
3D biomaterial and lyophilized kombucha beverage day 7 results.

Lyophilized black tea kombucha beverage and 3D biomaterial was shown according to the analysis at the end of the 7th day 58% viability, while lyophilized oolong tea kombucha beverage and 3D biomaterial was shown 78% viability (Figure 4).

**FIGURE 5:**
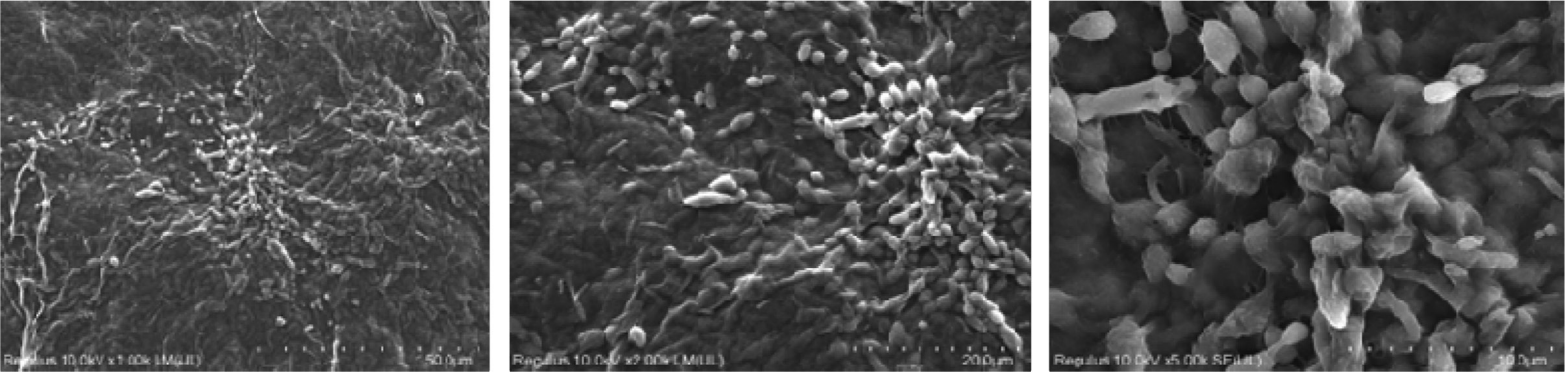
Seventh day SEM results of lyophilized oolong kombucha beverage and 3D biomaterial cell culture.

**FIGURE 6:**
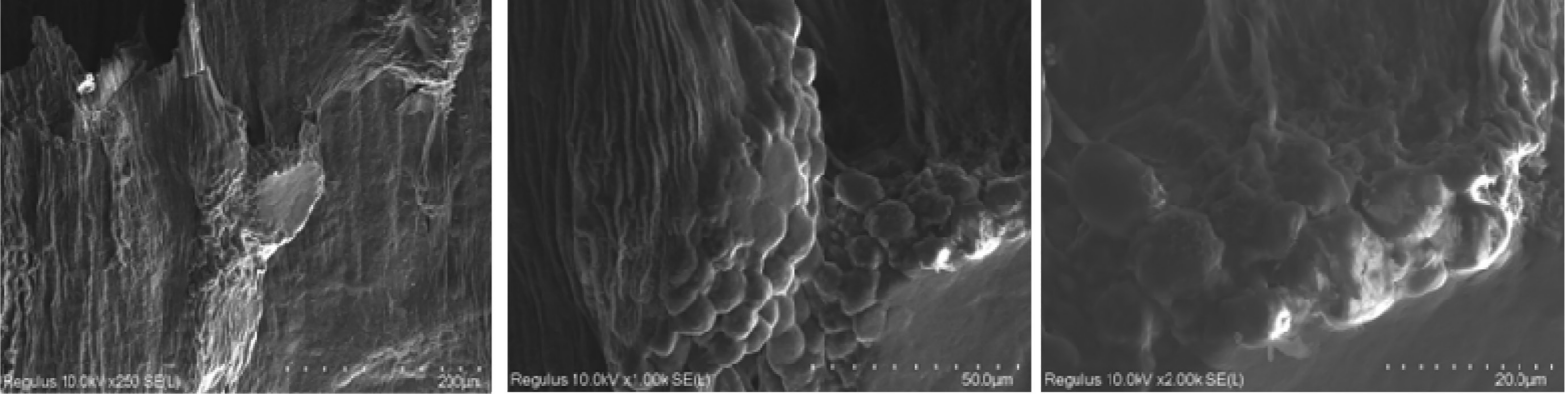
Seventh day SEM results of lyophilized black kombucha beverage and 3D biomaterial cell culture.

**FIGURE 7:**
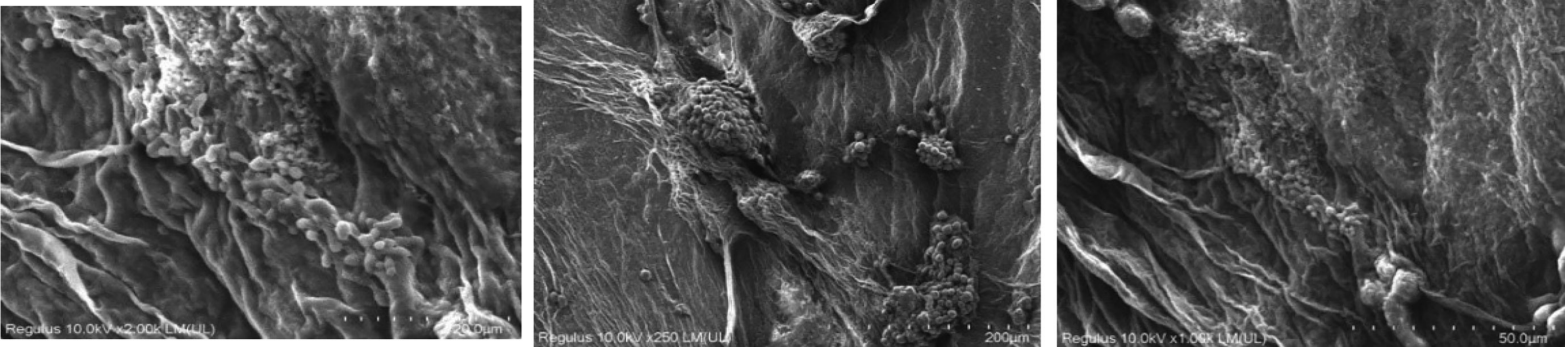
Twenty‐first day SEM results of oolong tea 3D biomaterial cell culture.

**FIGURE 8:**
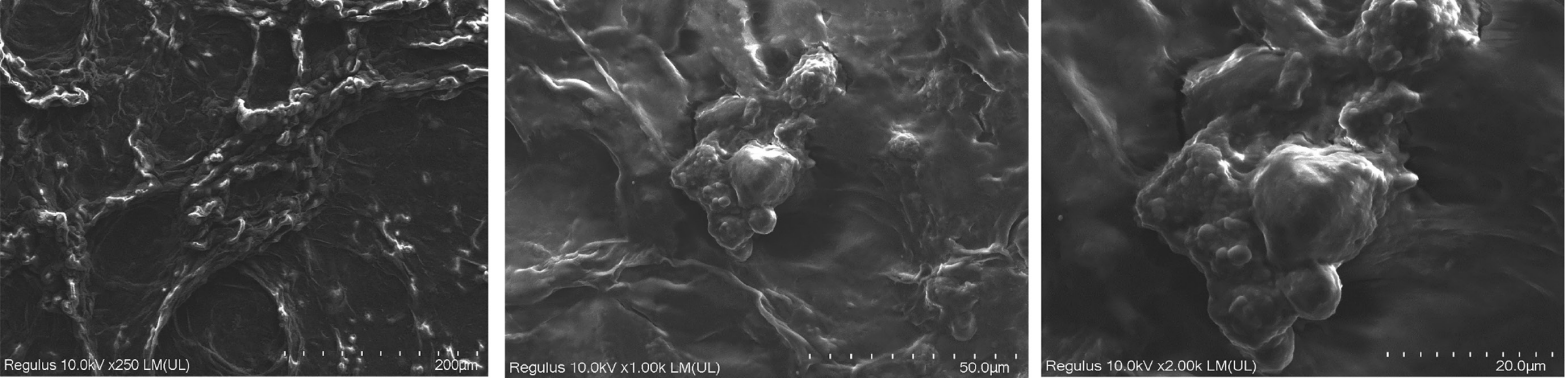
Twenty‐first day SEM results of black tea 3D material cell culture.

Figures 5, 6, 7, 8 show the SEM results of the studied samples. When the SEM results were evaluated, it was seen that fibroblasts provided adhesion to both oolong tea and black tea 3D Scaffold (Tissue Scaffold). It was concluded that adhesion is better in oolong tea. This result is compatible with cell culture results. When all the results were evaluated, it was concluded that the usage of both oolong tea and black tea biomaterials as 3D Scaffold (Tissue Scaffold) may be appropriate.

## 4 DISCUSSION

On the 21st day of fermentation, the pH of oolong tea kombucha beverage was 2.94; The pH of black tea kombucha beverage was measured as 2.88. As the fermentation period progresses, the amount of acetic acid in the medium increases and therefore becomes more acidic. In this study, as the fermentation period, it was preferred to end on the 21st day in order for the biofilms to reach sufficient thickness. In the study by Güldane et al. and by Miranda et al., they reported that as the fermentation time extended, the acidity level increased up to certain values and the pH value decreased.^7^, ^18^ The pH values in this study are compatible with the literature.

As in the study of Pillai et al. in 2021, a printable biomaterial suitable for gel‐like extrusion has been obtained in this study. Partial hydrolysis results of kombucha biofilms obtained as a result of fermentation are compatible with the results in the literature.^19^ Since it is not possible to obtain standard substances in HPLC analysis results of kombucha beverages, it could not be evaluated in terms of the components in the beverage, but kombucha beverages obtained with various teas in the literature were compared with HPLC analyses. The HPLC chromatogram of the black tea kombucha beverage they found according to the analysis conditions in the study of Miranda et al. is similar to the HPLC analysis chromatogram of the black tea kombucha beverage performed in this study.^18^ The reason for the different chromatograms is the analysis conditions and differences in fermentation times. Differences observed between HPLC chromatograms of oolong tea and black tea kombucha beverages indicate that there may be differences in composition depending on the fermentation source. 3D Scaffold (Tissue Scaffold) created from Kombucha Biofilms “L929 cell line (mouse fibroblast)” culture according to the analysis results on the 14th day, black tea showed 59% viability, while on the 21st day black tea showed 51% (Figure 2) viability. When these results are evaluated, it can be concluded that although there is a decrease in the number of viable cells compared to the control group, the cells maintain their viability to a large extent in the 3D Scaffold (Tissue Scaffold) medium. On day 14, oolong tea showed 74% viability. At the end of the 21st day, oolong tea showed 73% viability according to the analysis (Figure 2). It appears that the viability rate of oolong tea 3D Scaffold (Tissue Scaffold) is higher than that of black tea 3D Scaffold (Tissue Scaffold). It was concluded that Oolong tea 3D Scaffold (Tissue Scaffold) is more stable and more compatible in terms of biocompatibility since fibroblast cell viability does not decrease over time. While the IC_50_ value for the lyophilized form of kombucha from black tea on fibroblast cells was 6.05 mg, the IC_50_ value for the lyophilized form of kombucha from oolong tea was 7.53 mg (Figure 3). In light of these results, lyophilized kombucha beverages from oolong tea was found to be less cytotoxic at a semi‐lethal dose than lyophilized kombucha beverages from black tea. Lyophilized and 3D scaffold fibroblast cell cultures showed 58% viability for black tea at the end of the 7th day, while oolong tea showed 78% viability (Figure 4). It was concluded that oolong tea 3D Scafold (Tissue Scaffold) provided 20% more viability on fibroblasts. In addition, it can be stated that the addition of lyophilized tea can be effective in increasing fibroblast viability rates.

In this study, we first discuss fibroblast cell culture with 3D Scaffold (Tissue Scaffold) Created from Kombucha Biofilms. In our results, 3D Scaffold (Tissue Scaffold) created from Kombucha Biofilms “L929 cell line (mouse fibroblast)” culture according to the analysis results on the 14th day, black tea showed 59% viability, while on the 21st day, black tea showed 51% viability. When these results are evaluated, it can be concluded that although there is a decrease in the number of viable cells compared to the control group, the cells maintain their viability to a large extent in the 3D Scaffold (Tissue Scaffold) medium. On day 14, oolong tea showed 74% viability. At the end of the 21st day, oolong tea showed 73% viability according to the analysis. Kombucha natural polymers are homogeneously dispersed. In addition, the fibroblast culture tended to grow within itself and we believe that the possibility of tuning the mechanical properties of especially oolong 3D scaffolds to increase the collagen concentration is interesting. One study showed that in the cell scaffold created with bacterial nanocellulose fibres, stiffness increased by 43% while collagen retained its appropriate viscoelastic properties. Also, in the cell scaffold created with bacterial nanocellulose fibres, stiffness increased by 43% while collagen retained its appropriate viscoelastic properties. Moreover, in this study, the increased stiffness obtained in the cell scaffold formed with Bacterial nanocellulose fibres showed considerable stability due to the fact that type I collagen remained the main component. In our study, we think that the collagen content increases in 3D‐created bacterial cell scaffolds.^20^ In another study, it was commented that elucidating the cross‐link structure used by bacteria and the cross‐link formation mechanism in experiments conducted on the bacterial cytoskeleton is expected to facilitate synthetic biology using bacterial cytoskeletons. In our experiments, we think that the kombucha biofilm structure supports this.^21^ Our results showed that the viability rate of oolong tea 3D Scaffold (Tissue Scaffold) is higher than that of black tea 3D Scaffold (Tissue Scaffold). It was concluded that Oolong tea 3D Scaffold (Tissue Scaffold) is more stable and more compatible in terms of biocompatibility since fibroblast cell viability does not decrease over time. While the IC_50_ value for the lyophilized form of kombucha from black tea on fibroblast cells was 6.05 mg, the IC_50_ value for the lyophilized form of kombucha from oolong tea was 7.53 mg. In light of these results, lyophilized kombucha beverage from oolong tea was found to be less cytotoxic at a semi‐lethal dose than lyophilized kombucha beverages from black tea. Lyophilized and 3D scaffold fibroblast cell cultures showed 58% viability for black tea at the end of the 7th day, while oolong tea showed 78% viability. It was concluded that oolong tea 3D Scafold (Tissue Scaffold) provided 20% more viability on fibroblasts. In addition, it can be stated that the addition of lyophilized tea can be effective in increasing fibroblast viability rates.

SEM results showed that fibroblasts adhered to both oolong tea and black tea 3D Scaffold (Tissue Scaffold). It was observed that the cells established an integrity with the scaffold structure and formed the extensions required by a 3D structure. SEM images also look better in oolong tea than in cell culture results. In the Figure 8 sample, cell proliferation and extensions appear more prominent than the other samples. According to both SEM and cell culture results, it was concluded that adhesion was better in oolong tea. This result is consistent with the cell culture results. When all the results are evaluated, it is concluded that both oolong tea and black tea food biomaterials can be used as 3D Scaffold.

It will be investigated whether these findings obtained from in vitro methods can be used as biomaterials in our in vivo osteoarthritis pre‐clinical study.

## 5 CONCLUSION

We concluded that oolong tea kombucha 3D biomaterial is more suitable in terms of biocompatibility. Oolong tea kombucha 3D tissue scaffold can be considered as a more suitable potential candidate in bioimplant implementation.

## AUTHOR CONTRIBUTIONS

**İlkyaz Bağlan:** Conceptualization (equal); data curation (equal); funding acquisition (equal); investigation (equal); methodology (equal); project administration (equal). **Edagül Yanbakan:** Conceptualization (equal); data curation (equal); funding acquisition (equal); investigation (equal); methodology (equal); project administration (equal). **Tuğba Tuncel:** Conceptualization (equal); data curation (equal); funding acquisition (equal); investigation (equal); methodology (equal); project administration (equal); resources (equal); supervision (equal); writing – original draft (equal); writing – review and editing (equal). **Ayşe Koçak Sezgin:** Investigation (equal). **Emirhan Bozoğlan:** Investigation (equal). **Derya Berikten:** Investigation (equal). **Fatih Kar:** Conceptualization (equal); investigation (equal); methodology (equal); writing – original draft (equal); writing – review and editing (equal).

## CONFLICT OF INTEREST STATEMENT

The authors declare that there are no conflicts of interest.

## DEDICATION

As scientists raised in Turkey, we dedicate this publication to the 100th anniversary of the Republic of Turkey.

## Data Availability

The data sets generated during and/or analysed during the current study are available from the corresponding author upon reasonable request.
